# Tailorable Piezoelectric Chain Morphology in Biocompatible Poly‑l‑lactide Induced by Melt-Based 3D Printing

**DOI:** 10.1021/acsapm.5c00450

**Published:** 2025-05-06

**Authors:** Cristina Pascual-González, Gustavo Pacheco-Carpio, Juan P. Fernández-Blázquez, María Concepción Serrano, Bernd Wicklein, Miguel Algueró, Harvey Amorín

**Affiliations:** † Instituto de Ciencia de Materiales de Madrid (ICMM), CSIC, C/Sor Juana Inés de la Cruz 3, Cantoblanco, 28049 Madrid, Spain; ‡ IMDEA Materials Institute, C/Eric Kandel 2, Getafe, 28906 Madrid, Spain

**Keywords:** poly-l-lactide, crystallinity, polymer orientation, piezoelectricity, fused deposition modeling

## Abstract

Biobased and biodegradable poly-l-lactide (PLLA) stands out among piezoelectric polymers for its biocompatibility and environmental sustainability. Its piezoelectric response is closely related to the crystallinity and the alignment of polymer chains, which is conventionally obtained by drawing techniques. These are two-step processes with tight shape constraints, and the material technology implementation would strongly benefit from the demonstration of a single-step process capable of directly achieving tailored piezoelectric morphology in PLLA biopolymer from polymer melt. Fused deposition modeling (FDM) three-dimensional (3D) printing can play this role, directly achieving tailored piezoelectric morphology in PLLA biopolymer by the microscale control of molecular chain orientation through preparation parameters, such as 3D printing speed or bed temperature. The printing-crystal phase content and texture-piezoelectric property relationships are comprehensively presented, and the key 3D printing parameters to obtain optimized piezoelectric chain morphologies are defined. Results reveal melt-based 3D printing to be a suitable technique for manufacturing biocompatible PLLA piezoelectric platforms that are also biodegradable. A commercial PLLA (molecular weight of 160 kDa) has been used, with which a large shear piezoelectric coefficient (*d*_14_ = 8.5 pC/N) was attained after optimized printing. Biocompatibility *in vitro* with murine L929 fibroblasts is confirmed for this specific material, opening its use not only for smart monitoring but also for biomedical applications, including tissue engineering.

## Graphical abstract

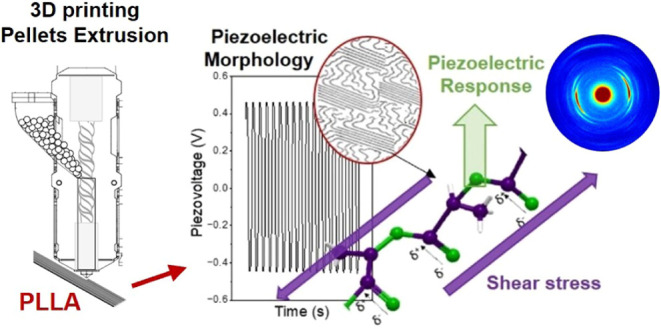

## 1 Introduction

There is an increasing demand for intelligent sensing systems due to the growing need for accurate data collection in various sectors such as healthcare, environmental monitoring, and smart cities.^1,2^ Indeed, implantable bioelectronic systems that continuously monitor physiological functions and simultaneously provide personalized therapeutic solutions for patients are required in many applications ranging from neural systems to bioelectronic organs.^3^ In parallel, and in order to mitigate the increasing electronic waste associated with the massive expansion of sensors and other electronic devices, responsible electronics (also green electronics) have gained relevance in recent years. This aims to pave the way for a greener future, where technology and sustainability coexist.^4^ In this context, polymers are bound to play a crucial role in the advancement of responsible electronics, offering benefits that align with the required goals of sustainability, cost-efficiency, and innovation in design. However, there is still a long way ahead, where a comprehensive assessment thoroughly examining the practical implementation of biopolymer sensors is mandatory.

Piezoelectric materials are a key enabling technology for the next generation of flexible smart devices, with great prospective in the biomedical field.^5^ When a piezoelectric material is deformed under stress, an asymmetric displacement of charges occurs, resulting in a change in electrical polarization (a direct effect used in sensing and energy harvesting applications). Conversely, these materials also deform linearly under an electric field, which is used in turn for actuation and ultrasound technologies. Commercial state-of-the-art piezoelectric materials are ceramics (Pb­(Zr,Ti)­O_3_ and BaTiO_3_)^6^ that are brittle and stiff and cannot satisfy the increasing demand of flexible systems. Although polymers have a lower piezoelectric response, they are currently under study as they have many other advantages, such as easy processing, flexibility, and lightweight.^7,8^ The piezoelectric polymer per excellence today is poly­(vinylidene fluoride) (PVDF), in particular, the copolymer with trifluoroethylene PVDF-TrFE.^9^ However, these fluorinated polymers are neither biobased nor biodegradable, which are critical drawbacks for many applications like transient medical implants.^10^ The search for alternative, more sustainable piezoelectric polymers is an open challenge, where biopolyesters such as the poly-l-lactic acid (PLLA) are of growing interest due to their origin from renewable sources, biocompatibility, and biodegradability.^11^

PLLA is a versatile thermoplastic polymer that has been widely applied in packaging and textiles^12,13^ and it is also proposed for biomedical applications due to its ability of being absorbed in the human body.^14–16^ Piezoelectric response in PLLA is achieved when polymer crystals become highly oriented (previously named as piezoelectric morphology^17^) during the fabrication process, typically by cold drawing,^14,18^ and thought to be associated with the polarity induced by the reorientation of carbonyl groups.^11^ Note that an electrical poling treatment is not necessary, unlike that for PVDF, which would simplify the manufacturing of polymer-based piezoelectric devices. Stretching and cold-drawing techniques are traditional methods to induce piezoelectric properties in PLLA, for which the shear piezoelectric coefficient *d*_14_ ≈ 10 pC/N is typically reported.^19^ Although this reference is more than 20 years old, it remains a highly cited work regarding the piezoelectric characterization of PLLA.^20^ More recently, Yoshida et al.^21^ reported values up to 20 pC/N for films processed by solid-state extrusion, as well as Ben Achour et al.^22^ that measured 10–20 pC/N depending on processing conditions. However, these techniques are not suitable for the fabrication of piezoelectric PLLA-based devices, since they do not allow the control of shape and dimension nor produce complex geometries.

In recent years, other fabrication techniques such as electrospinning have been demonstrated as effective means to produce highly oriented PLLA fibers with piezoelectric chain morphology.^14,23–26^ In spite of the good performance of the piezoelectric electrospun fibers, for which *d*_14_ values above 10 pC/N were roughly estimated by impact measurements,^14^ and the advantages of the technique, namely, making a poststretching treatment unnecessary, this technique implies the use of toxic solvents that must be carefully removed after fabrication. More recently, a piezoelectric PLLA fiber with a high degree of crystallinity and lamellae orientation has been obtained by a melt-spinning technique.^17^ A single-step process from the melt, instead of the usual two steps (one for elongation of the fiber from the melt and a second one for hot drawing of the solidified fiber), was used in this case. This is a distinctive advantage for the large-scale production of flexible piezoelectric films.

Another promising group of technique are three-dimensional (3D) printing technologies, which have emerged as alternative approaches for the production of biobased electronic components, capable of realizing complex geometries and of fabricating structures with high spatial resolution and accuracy.^27,28^ Among 3D printing techniques, stereolithography,^29^ selective laser sintering,^30^ inkjet printing,^31^ and extrusion-based methods^32^ have been successfully applied to piezoelectric polymers. Fused deposition modeling (FDM) is one of the extrusion methods that involves the melting of a thermoplastic polymer with a moving extruder and depositing the molten material layer-by-layer, following a 3D model. Simultaneous application of the electric field is possible, and FDM has been successfully used to obtain piezoelectric PVDF-based composites where an *in situ* poling was introduced directly from printing.^33,34^ Although a literature review confirms the 3D printing potential for optimizing the resolution, dimensions, fabrication time, and costs of the final products, a sound understanding of how the 3D printing parameters determine electromechanical performance is missing.

This is especially true for PLLA,^28,35^ for which several works have pointed out that 3D-printed structures can show certain chain orientation, yet they are usually amorphous due to the slow crystallization rate characteristic of the biopolymer.^22,36^ Postprinting *in situ* annealing becomes thus necessary to increase crystallinity and obtain a sizable piezoelectric response, although this is limited by misorientation of the chains due to thermal relaxation. Nonetheless, Hayashi et al. succeeded in manufacturing a piezoelectric PLLA smart phone case by FDM^32^ and suggested a role of printing speed during FDM in the functional response, yet reported *d*_14_ values were rather small (<2 pC/N). This is the only precedent of piezoelectric PLLA fabricated by 3D printing, and there is a distinctive lack of understanding of how printing parameters control the degree of crystallinity, crystal phase, and chain orientation that ultimately determine the piezoelectric response.

This work discloses the relationships among 3D printing parameters, crystallinity, and crystalline orientation, and piezoelectric response of PLLA biopolymer, whose knowledge is essential to produce high piezoelectric response platforms that enable biocompatible piezoelectric devices, which are also biodegradable, to be obtained. Additionally, FDM presents different advantages to induce piezoelectric properties in PLLA over the other techniques mentioned above. First, it is a solvent-free method and, therefore, a more environmentally friendly approach. FDM is also a widely used and cost-effective technology, which increases its potential for automation and industrial scalability. Finally, as an advanced additive manufacturing technique, melt-based 3D printing allows the fabrication of complex and customized structures.

## 2 Methods

### 2.1 Material and 3D Printing

The novelty of this equipment lies in its capability to work with materials that are not in a filament format. A set of six samples were 3D-printed under variable parameters from a commercial PLLA (Ingeo Biopolymer 6202D, molecular weight of 160 kDa, NatureWorks LLC), a thermoplastic fiber-grade resin derived from renewable resources, and analyzed in this work. Layer height (0.2 mm) and nozzle diameter (0.4 mm) were the same for all samples. Figure 1 summarizes the ensemble of 3D printing parameters investigated. The objective was to establish the effect of 3D printing speed (*v*_3D_), bed temperature (*T*_B_), and postprocessing treatment (i.e., an annealing at temperatures between the glass transition and melting) on the degree of crystallinity, crystal phase, and crystallite alignment of the PLLA layers and to correlate them with the piezoelectric response, in order to define the conditions for directly obtaining controlled piezoelectric morphologies in PLLA by FDM. Samples 1 and 2 were 3D-printed by keeping the printing bed at room temperature (RT). The rapid cooling of molten material in contact with the print bed is expected to hinder the crystallization of PLLA, which means that these samples would remain in an amorphous state after 3D printing. The effect of a high printing speed on the characteristics of the amorphous phase is analyzed in sample 2.

**Figure 1:**
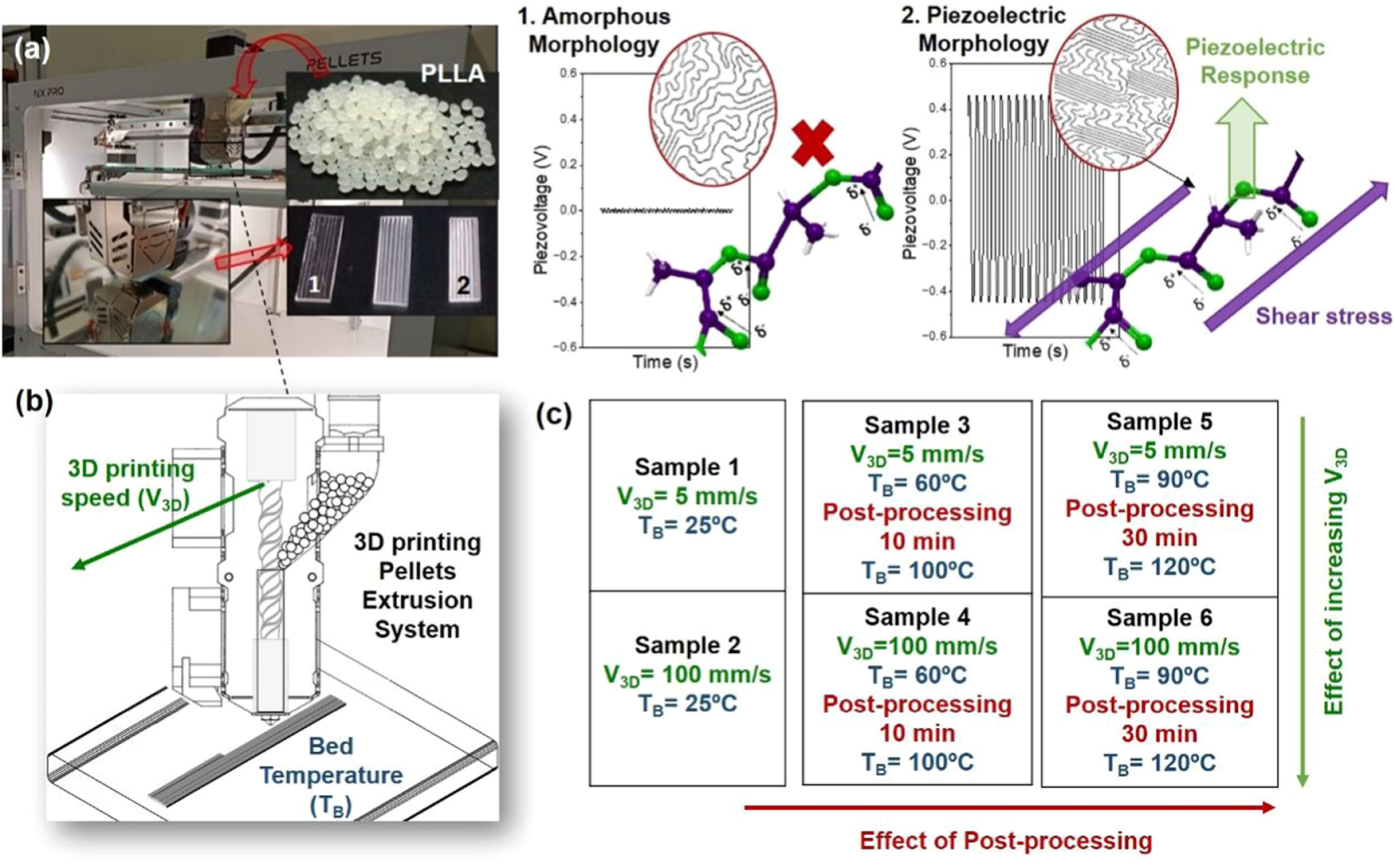
(a) Schematic overview of the study: commercial PLLA granulate feeds the 3D printing pellet extrusion system; different polymer morphologies can be obtained from the same material by modifying 3D printing parameters; piezoelectric morphology term is used to summarize the characteristics to obtain the piezoelectric response from PLLA (highly oriented crystals). (b) Illustration of the pellet extrusion system used in this work (FDM technology). (c) Summary of the ensemble of 3D-printed samples and their corresponding experimental conditions.

Parameter modifications in samples 3–6 were proposed based on the calorimetric properties of commercial PLA,^37^ with the aim of increasing crystallization of printed PLLA through different mechanisms. First, and in order to promote the crystallization from melt, the cooling rate of the molten material when it comes in contact with the printing platform has to be significantly reduced. This was attained here by increasing the bed temperature during the printing process, which was set between the glass transition of PLLA, which is usually reported between 55 and 60, and 100 °C where cold crystallization PLLA starts, as observed by differential scanning calorimetry (DSC).^37^ The adhesion on the printing bed and the diffusion of the molten material are ensured in this temperature range without overpassing the temperature of cold crystallization. Samples 3 and 4 were printed on the bed at 60 °C and samples 5 and 6 at 90 °C. Second, once the samples were 3D-printed, the bed is further heated and maintained isothermally for different times to promote cold crystallization. The temperatures were chosen for low (100 °C) and high crystallization (120 °C). Samples 3 and 4 were treated on the printing bed at 100 °C for 10 min, while samples 5 and 6 were treated at 120 °C for 60 min.

It is important to bear in mind that PLLA can adopt different crystalline phases (polymorphs) that are very sensitive to processing conditions, which in turn has been reported to contribute distinctly to the piezoelectric behavior.^38^ An ordered α-phase is typically produced at high crystallization temperature, where helical chain segments are assembled into an orthorhombic unit cell, while a less ordered α′-phase usually crystallizes at about cold crystallization temperature.^39,40^ Regarding the β-phase, first time obtained in stretched fibers,^41^ it is believed to be an intermediate phase of the low crystallization temperature α′-PLLA variant. In all cases, the piezoelectric activity of PLLA is intrinsically linked to the chain orientation, so crystal alignment is necessary. This requires the molten material that is extruded through the nozzle to be subjected to high elongation rates. Therefore, the effect of increasing 3D printing speed from 5 to 100 mm/s (shear rate at the nozzle of 12.5 and 250 s^–1^, respectively) on the degree of crystal orientation and piezoelectric response was also evaluated. Indeed, the optimization of 3D printing parameters aims to maximize both crystallinity and molecular orientation of PLLA. The optimized sample refers to sample 4 with a longer annealing time (30 min) at 100 °C in order to increase crystallinity while preventing chain relaxation.

### 2.2 Physicochemical Characterization

Samples were extracted from the 3D-printed 150 mm × 100 mm laminates for the characterization, which had a thickness ranging from 70 to 150 μm depending on processing parameters. The crystallinity of the 3D-printed samples was studied by differential scanning calorimetry (DSC) using a Q200 instrument from TA Instruments. 5–10 mg samples were heated from 0 to 180 °C at a heating rate of 10 °C/min in a N_2_ atmosphere. The thermograms provide the glass-transition temperature (*T*_g_), cold crystallization temperature (*T*_cc_), and melting temperature (*T*_m_), so that information on how these temperatures change with 3D printing parameters can be obtained. Besides, the degree of crystallinity is estimated as the ratio between the melting enthalpy (Δ*H*_m_) and the theoretical value Δ*H*_o_ for 100% crystalline PLLA (the value of 93.6 J/g is widely accepted^42^), where Δ*H*_m_ is obtained by subtracting the cold crystallization enthalpy (Δ*H*_cc_) from the experimental endothermic enthalpy at *T*_m_. It is worth mentioning that the thermodynamic properties of PLLA in the literature are quite discordant, and values reported for Δ*H*_o_ actually range between 82 and 203 J/g.^43^ Indeed, the value of 93.6 J/g, recurrently assumed for piezoelectric PLLA, has been reported for d l-lactide copolymers.^44^ Nevertheless, for the sake of comparison with the previous literature, it is used here once more.

The crystal phases of 3D-printed laminates (5 mm × 5 mm samples) were determined by wide-angle X-ray scattering (WAXS) in SAXSpoint 5.0 (Anton Paar), equipped with a Cu Kα microfocus source (λ = 1.5418 Å) and using a two-dimensional Pilatus 1 M (Dectris) detector that allows us to analyze the preferred directions in the formation of polymer crystals. 2D patterns were converted into one-dimensional (1D) intensity profiles using the SAXS analysis software. The degree of molecular orientation was estimated from the azimuthal intensity distribution I­(φ) of the main diffraction ring at 2θ ∼ 16.7°, which represents the (200)/(110) reflection of α-phase PLLA. The azimuthal intensity profile is used to calculate the Hermans orientation factor (*f*_c_), which is 0 for a fully isotropic structure and 1 for a perfect uniaxial orientation, as explained elsewhere.^41,45^ The direction of the detector parallel to the 3D printing direction was defined as φ = 0.

Silver electrodes were deposited by sputtering on both sides of 3D-printed PLLA laminate samples for electrical and electromechanical characterizations. The temperature and frequency dependences of dielectric permittivity and losses were studied by complex impedance analysis using an HP4284A precision LCR Meter (Agilent) and a Janis VPF-700 Cryostat coupled to a temperature controller (Lakeshore model 331). Measurements were dynamically carried out during heating at 2 °C/min from −25 °C up to 125 °C. The piezoelectric characterization of the 3D-printed laminates was carried out on samples of 20 mm length and 5 mm width (electrode area) by using a universal dynamic testing system (Shimadzu model MMT-101NV-10) equipped with a load cell of 100 N, which allows *in situ* measurements of the piezovoltage response under stretching cycles at frequencies between 0.1 and 100 Hz (see Supporting Figure S1).

The open-circuit voltage was first measured using a potentiostat (PalmSens4 impedance analyzer) directly connected in parallel to the samples, while the response in short-circuit was also measured by collecting the piezoelectric charges generated using a charge-to-voltage converter in charge mode operation (TE Connectivity model Piezo Lab Amplifier). It should be mentioned that PLLA exhibits shear piezoelectric response (nonzero coefficients are *d*_14_ and *d*_25_ = −*d*_14_), where 1 corresponds to the direction of the generated electric field (or voltage) and 4 to the shear stress applied (a right-handed rotation around axis 1) in the Voigt notation.^20^ Thus, when a molecular chain is sheared along its axis, an electrical polarization develops in the direction perpendicular to the shearing plan.^46^ In practice, an “effective” transverse piezoelectric coefficient *d*_31_* can be measured if samples with printing fibers rotated 45° from the loading direction are tensile strained, from which *d*_14_ can be obtained as *d*_14_ = 2 *d*_31_* (detailed matrix transformation can be found elsewhere).^19,22^ Therefore, the piezoelectric characterization was carried out on PLLA rectangular samples cut at 45° in relation to the stretching axis,^47^ in our case the printing direction.^22^ In the experiments, the force amplitude (*F*) is increased from 1 to 5 N, while the charge density (*D*) generated is measured and the shear piezocoefficient *d*_31_* calculated from its linear dependence on the applied stress (σ)

1 $$d_{31}^{*}=\frac{D}{\sigma }$$

where

2 $$\sigma =\frac{F}{t\cdot w}\;\mathrm{and}\;D=\frac{Q}{l\cdot w}$$

and *t* is the thickness, *w* is the width, and *l* is the length of the 45°-cut PLLA samples (see Supporting Figure S1).

### 2.3 Biological Characterization

A fiber-grade commercial PLLA was used, and biocompatibility *in vitro* of the resulting piezoelectric 3D-printed PLLA laminates was assessed with murine L929 fibroblasts (ATCC). Prior to cell culture, samples were sterilized under ultraviolet (UV) radiation in a biological safety cabinet (30 min). Cell seeding density was 20,000 cells/well on 24-well plates containing the square-shaped PLLA samples. Cells were maintained in complete Dulbecco’s modified Eagle’s media (DMEM) supplemented with fetal bovine serum (10%), streptomycin (100 UI/mL), penicillin (100 UI/mL), and GlutaMAX (1%) for up to 7 days *in vitro* in a sterile incubator with 5% CO_2_, 37 °C, and a humid atmosphere.

For morphological examination, cells cultured on PLLA samples were fixed with glutaraldehyde (2.5% in distilled water) for 45 min at room temperature and dehydrated in a series of ethanol with increasing concentrations (30, 50, 70, 90, and 100%) for 15 min each (twice). Once completely dried, samples were mounted on metal stubs using carbon tape, coated with a thin (8 nm) gold layer, and visualized by using an FEI VERIOS 460 scanning electron microscope (SEM). Cell viability was evaluated by using a Live/Dead kit according to the manufacturer’s instructions (Invitrogen), which is based on calcein (live cells emitting intense green fluorescence) and ethidium homodimer-1 (EthD-1; dead cells emitting intense red fluorescence). After incubation with the probes for 5 min at 37 °C, the samples were visualized with a Leica SP5 confocal laser scanning microscope (CLSM). The fluorescence of both probes was excited by an argon laser tuned to 488 nm. After excitation, emitted fluorescence was separated using a triple dichroic filter 488/561/633 and measured in the range of 505–570 nm for green fluorescence (calcein) and 630–750 nm for red fluorescence (EthD-1). ImageJ software was employed to quantify cell viability by measuring the area covered by green- and red-labeled cells, which were expressed as a percentage of the total image area.

### 2.4 Statistics

Biological values were expressed as the mean ± standard error of at least 3 independent experiments (*N* ≥ 3). In each experiment, samples were typically analyzed in duplicate, with at least 3 images per replicate. Statistical analysis was performed using the Statistical Package for the Social Sciences (SPSS, version 27.0, IBM). Comparisons with respect to the control groups were done by a *t* Student test. In all cases, the significance level was defined as *p* < 0.05.

## 3 Results

Figure 2 shows the first heating DSC curves of all samples, while the thermal properties obtained from the curves are summarized in Table 1. DSC curves of samples 1 and 2 (Figure 2a) first exhibit an endothermic peak at 53 °C corresponding to the glass transition, followed by the appearance of a small endothermic peak associated with polymer aging. At higher temperature, an exothermic process due to cold crystallization is observed starting above 100 °C, while the double endothermic transition above 150 °C corresponds to the polymer melting. The peak integration area associated with the cold crystallization is about the same as the polymer melting for both samples, indicating that they are nearly amorphous at RT (a crystallinity below 5% was calculated; Table 1). This confirms the hypothesis that forcing the molten material to fastly cool hinders the crystallization, which is typically associated with the slow crystallization rate of PLLA.^48^ Note that both materials exhibit *T*_g_ at about 53 °C, suggesting that the amorphous state is not affected by the 3D printing speed. Instead, the cold crystallization of sample 2 takes place at a lower temperature, indicating that the high 3D printing speed reduces the physical entanglements and increases the molecular chain orientation in the amorphous material, favoring nucleation and promoting cold crystallization at a lower temperature. This is in agreement with the trend observed in PLLA melt-spun fibers that showed a systematic decrease of *T*_cc_ with increasing elongation rate.^14,17^ Moreover, the double melting peak observed in both samples is characteristic of melting–recrystallization–melting processes, a phenomenon usually ascribed to the presence of some unstable crystals formed during cold crystallization that melt a bit earlier and immediately recrystallize.^49^ Figure 2b shows the heating DSC curves of samples 3 and 4, where similar profiles were also found, yet some differences with samples 1 and 2 are worth mentioning. First, the cold crystallization exothermic area decreased so that samples 3 and 4 are semicrystalline at RT with a crystallinity of about 26% and 33%, respectively (Table 1). Therefore, the reduction of the cooling rate of molten PLLA during 3D printing combined with the postprocessing annealing at a temperature near *T*_cc_ clearly promotes crystallization, which is also favored by using a high printing speed as in sample 4. Second, *T*_g_ increases up to 60 °C in both samples, slightly higher than samples 1 and 2, indicating the reduction of chain mobility in the amorphous phase with increasing crystallinity. Third, *T*_cc_ shifts toward lower temperatures compared to samples 1 and 2 (about 10 °C lower), demonstrating the nucleating effect of PLLA crystals on increasing the driving force for cold crystallization at lower temperature. Third, the double melting behavior is less prominent, although it is still noticeable, due to the lower amount of unstable crystals nucleated from cold crystallization, while *T*_m_ coincides with the second melting point of samples 1 and 2 (Table 1).

**Figure 2:**
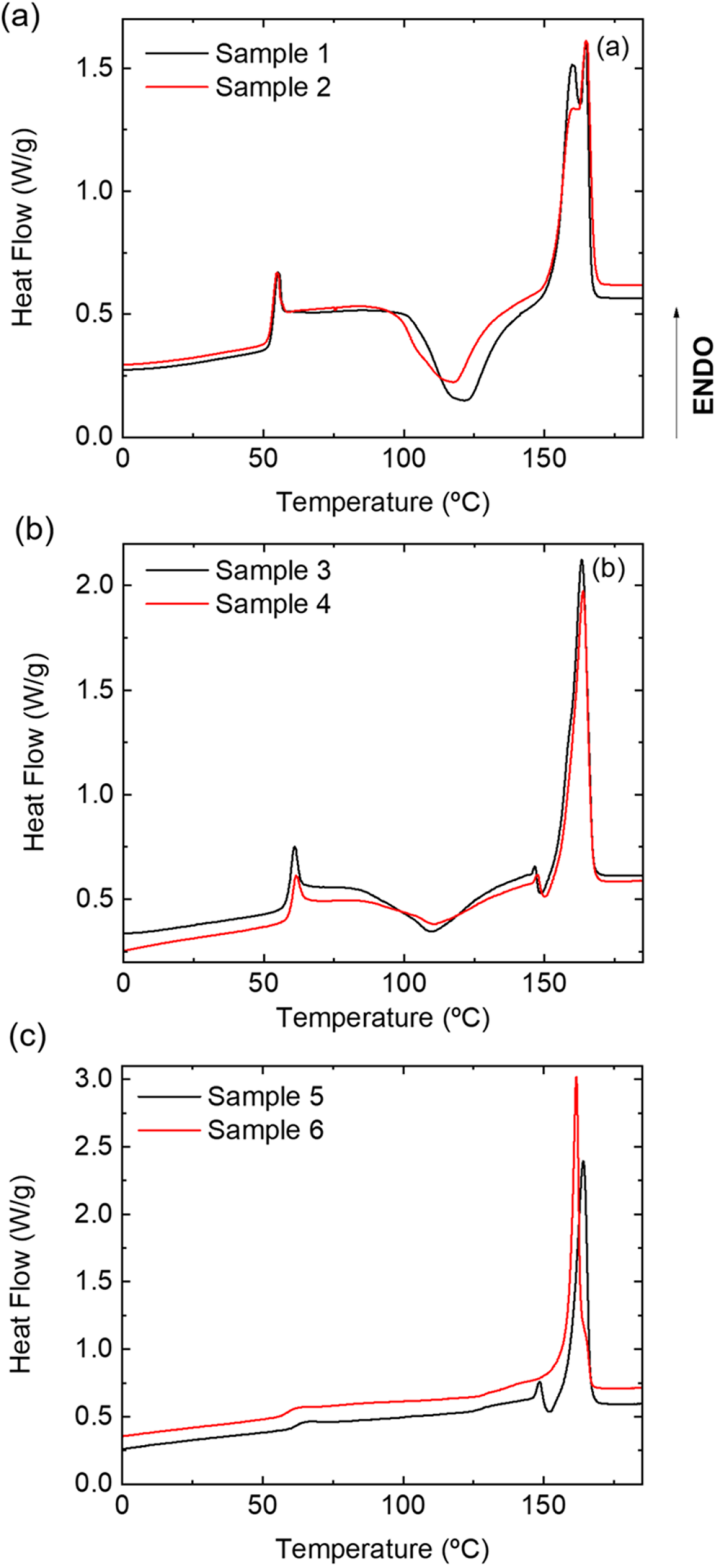
Heat flow curves during 1st heating at 10 °C/min for (a) samples 1 and 2, 3D-printed on a platform at RT at 5 and 100 mm/s, respectively; (b) samples 3 and 4, 3D-printed on a platform at 60 °C at 5 and 100 mm/s, respectively, and annealed at 100 °C for 10 min; and (c) samples 5 and 6, 3D-printed on a platform at 90 °C at 5 and 100 mm/s, respectively, and annealed at 120 °C for 30 min.

**Table 1:** Thermal Properties of the 3D-Printed PLLA Samples Obtained from the 1st Heating DSC Curves, Which Includes Melting Enthalpy (Δ*H*_m_), Degree of Crystallinity (*X*_c_), Glass-Transition Temperature (*T*_g_), Cold Crystallization Temperature (*T*_cc_), and Melting Points (*T*_m_)­^a^

sample	Δ*H*_m_ (J/g)	*X*_c_ (%)	*T*_g_ (°C)	*T*_cc_ (°C)	*T*_m_^1^ (°C)	*T*_m_^2^ (°C)
1	3.7	4.0	53.7	121.7	160.1	164.6
2	4.5	4.8	53.1	117.4	160.1	164.8
3	24.6	26.3	59.2	109.2		163.2
4	30.8	32.9	60.0	110.2		163.7
5	64.9	69.3	64.2			164.1
6	68.7	73.4	59.5		161.6	
optimized	54.8	57.1	61.7		158.7	162.4

a Moreover, the double melting peak observed in both samples (mentioned as T1m and T2m in table) is characteristic of melting–recrystallization–melting processes.

Note the small endothermic peak observed just below 150 °C followed by a small exothermic process in both samples, which seems to indicate a transition from α′- to α crystals. Indeed, similar anomalies in DSC curves just prior to melting peak have been usually ascribed to a first-order-type disorder-to-order (α′ to α) phase transition, related to α′ rearrangement upon approaching the melting point.^50–52^ The presence of α′ phase is the result of the postprocessing annealing at a temperature near cold crystallization, usually observed in PLLA after heating in the 70–110 °C range samples that had been quenched in the glassy state.^39^

Finally, the DSC results of samples 5 and 6 are given in Figure 2c. None exhibits the exothermic process associated with cold crystallization, beyond a very slight curvature above 100 °C, which means that the expected crystallization has already occurred during 3D printing and postprocessing, reaching a high degree of crystallinity in both samples of about 70% (Table 1). Again, the crystallinity of sample 6 is slightly higher, confirming the influence of using a high 3D printing speed. The DSC curve of sample 5 also shows the small anomaly associated with the transition from α′ to α crystals, analogous to samples 3 and 4, while sample 6 does not. Note also a single melting peak in both cases, although at different temperatures; while *T*_m_ of sample 5 matches the values of samples 3 and 4, sample 6 seems to indicate a different crystalline state, perhaps related to different polymorphs. Indeed, the appearance of double melting peaks has also been attributed to different crystalline structures.^53^

It can be, therefore, concluded that 3D printing parameters can be tuned to prepare semicrystalline PLLA laminates with tailorable degrees of crystallinity, up to values remarkably high and previously unrecorded by FDM, actually comparable to the best achieved ones by cold-drawing or melt-spinning techniques.^17^ In addition, the positive effect of using a high 3D printing speed on the kinetics of PLLA crystallization has been demonstrated. It is thought that the molten material subjected to high elongation rates reduces molecular chain entanglements and facilitates crystallization, so that the formation of different PLLA polymorphs is promoted.

Figure 3a–d shows the 1D-WAXS profiles of all samples. An amorphous halo with no trace of crystalline diffraction peaks is clearly observed for samples 1 and 2 in Figure 3a, consistent with DSC results that indicate a low degree of crystallinity (below 5%). 1D-WAXS patterns of samples 3 and 4 reveal the emergence of several peaks, as shown in Figure 3b, among which the strongest are (200)/(110) at 16.5° and (203) at 18.9°, which are typical reflections of α phase.^39,53,54^ However, note that scattering peaks for α-crystals are usually located at 16.7 and 19.1° (for the above-mentioned family of crystallographic planes) and a shift toward lower angles is usually associated with the coexistence of disordered α′ and α crystals. Note also that α′ presents the same crystal lattice as α-form but with a reduced chain packing.^55^ This seems to indicate that α′ is the polymorph at RT in these samples as a result of the postprocessing annealing performed, consistent with the transition from α′ to α-form observed in DSC curves. Nevertheless, there is still a significant amorphous halo in the patterns that does not allow one to distinguish small peaks for in-depth analysis of the actual phases. The higher intensity of the reflections of sample 4, in relation to the amorphous halo, is consistent with the higher crystallinity calculated from the DSC curves. On the other hand, 1D-WAXS patterns of samples 5 and 6 are given in Figure 3c, where several differences can be distinguished. First, the main peaks of sample 5 match exactly those appearing in patterns of samples 3 and 4, indicating the presence of α′-form at RT, also consistent with the observation of the transition from α′ to α crystals in the DSC curve. Indeed, reflection at about 24.7° (116) does not correspond to the pure α-form, as shown in Figure 3d, and has been identified with α′-form.^52,56^ However, the main peaks of sample 6 are slightly shifted toward higher angles, *e.g.*, to 16.7 and 19.1° for the above-mentioned reflections, which clearly indicates a denser chain packing and the presence of the more thermally stable α-form at RT in this sample.^39,53,54^ Besides, the DSC curve of sample 6 shows no evidence of the transition from α′ to α crystals, and the melting peak took place at a bit lower temperature (Table 1), highlighting the different behaviors of these two polymorphs. Note that there is a shoulder at a higher temperature of the melting point, which should be related to melting–recrystallization–melting processes of α crystals. Again, the higher intensity of side peaks for sample 6 is consistent with the higher crystallinity calculated from DSC curves. Note also that *T*_g_ of sample 6 was observed about 5 °C below that of sample 5, in agreement with reports on full crystallized α-form PLLA.^50^ Therefore, the slow cooling rate of sample 5 seems to favor the crystallization from the melt of α′ crystals that do not evolve during the annealing, while the high cooling rate of sample 6 allows the material to evolve to stable α-phase during the annealing.

**Figure 3:**
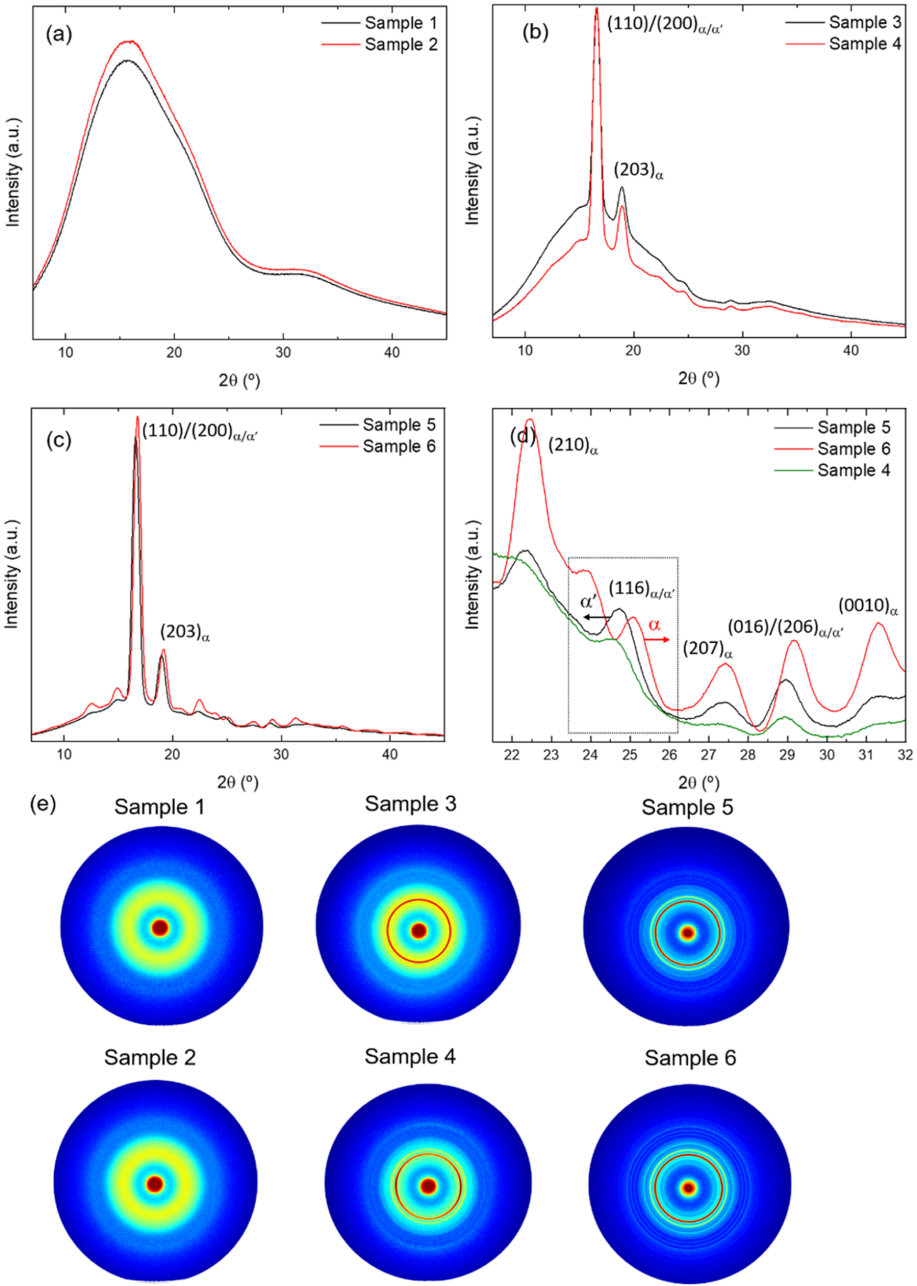
1D-WAXS diffraction patterns corresponding to (a) samples 1 and 2, 3D-printed on a platform at RT at 5 and 100 mm/s, respectively; (b) samples 3 and 4; 3D-printed on a platform at 60 °C at 5 and 100 mm/s, respectively, and annealed at 100 °C for 10 min; (c) samples 5 and 6; 3D-printed on a platform at 90 °C at 5 and 100 mm/s, respectively, and annealed at 120 °C for 30 min; and (d) closer inspection of 1D-WAXS diffraction patterns of samples 4–6. The inset dashed box highlights the peak (116), which confirms the presence of α′ in samples 4 and 5. (e) 2D-WAXS patterns for all six 3D-printed samples. Peaks corresponding to α and α′ phases are indicated.

Most reports have focused on the control of crystallinity through 3D printing.^57,58^ However, only a few studies have investigated both the crystalline structure and orientation of PLLA through additive manufacturing techniques.^59^ 2D-WAXS patterns of all samples are given in Figure 3e. The general observation agrees well with the 1D-WAXS patterns. Samples 1 and 2 have the typical amorphous halo with a wide scattering peak at 2θ ∼ 15°, which is still visible in samples 3 and 4, though concentric diffraction rings start emerging in these samples associated with α′-crystals. Note, however, that the most intense ring, associated with the principal (200)/(110) reflection, changes from highly isotropic in sample 3 (indicative of randomly oriented crystals) to a slightly arc-like ring in sample 4, which means a preferred crystal orientation. Finally, narrower concentric rings of higher intensity are obtained in samples 5 and 6, where the amorphous halo decreases significantly, indicating a higher crystallinity, as calculated from DSC results. Noteworthy, the principal diffraction ring is highly isotropic in both samples, suggesting they are randomly oriented, unlike sample 4.

The azimuthal intensity profiles for the strongest ring (ascribed to the (200)/(110) reflection) of all samples are shown in Figure 4, which are used to calculate the Hermans crystal orientation, given in Table 2 (peak analysis for *f*_c_ calculation is displayed in Figures S2–S4). Note that sample 2 reveals two tiny but sharp peaks yielding a *f*_c_ = 0.95, indicating that at the early stage of crystallization (below 5% in this sample) regions with highly preferential chain orientation crystallizes. Figure S5 shows an incipient crystallization of sample 2 by integrating a smaller area of 2D-WAXS pattern. A preferred crystal orientation is also observed in sample 4, which showed higher crystallinity with moderate *f*_c_ = 0.54 induced by the high 3D printing speed used as compared to sample 3, which is essentially randomly oriented. Moreover, samples 5 and 6 do not show any crystal orientation, as no peaks are observed in the azimuthal profiles. It is evident that increasing the postprinting temperature from 100 to 120 °C promotes further polymer chain relaxation and thus favors isotropization.

**Figure 4:**
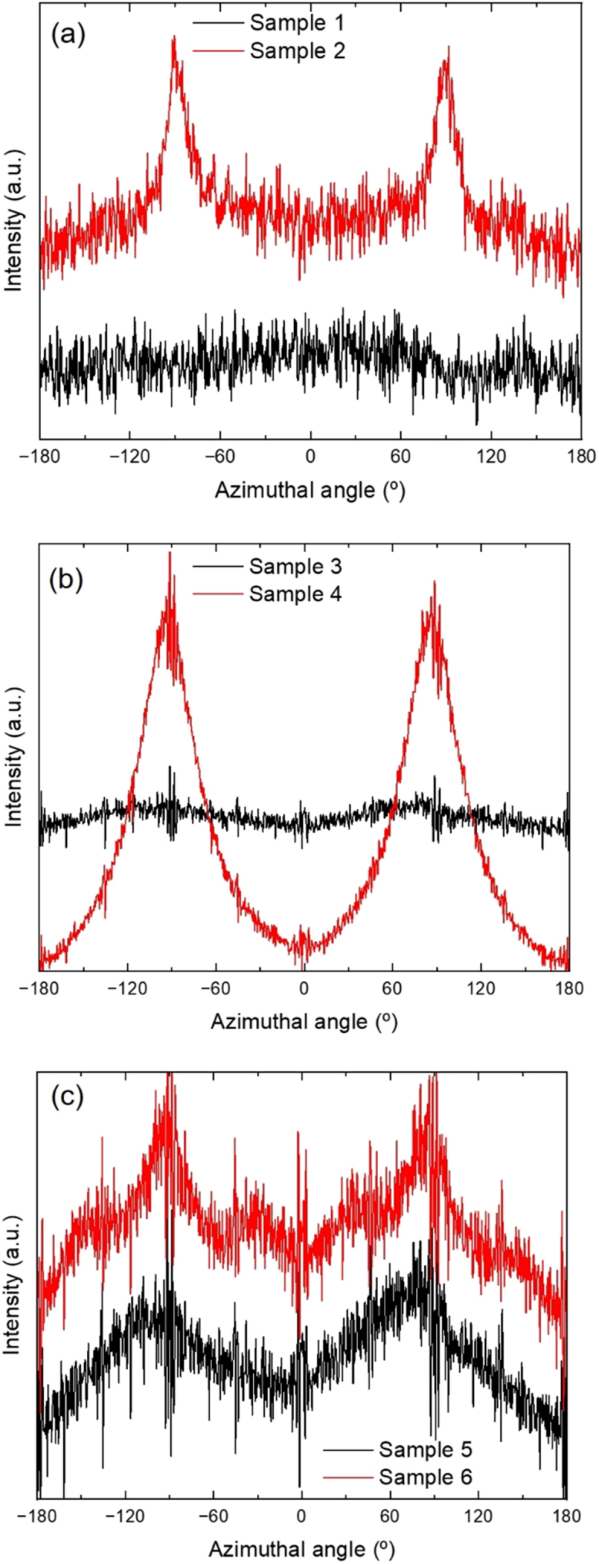
(a) WAXS azimuthal intensity profiles of samples (a) 1 and 2, 3D-printed on a platform at RT at 5 and 100 mm/s, respectively; (b) samples 3 and 4, 3D-printed on a platform at 60 °C at 5 and 100 mm/s, respectively, and annealed at 100 °C for 10 min; and (c) samples 5 and 6, 3D-printed on a platform at 90 °C at 5 and 100 mm/s, respectively, and annealed at 120 °C for 30 min.

**Table 2:** Crystallinity (*X*_c_), Hermans Orientation Factor (*f*_c_), Dielectric Permittivity (ε), and Shear Piezoelectric Coefficient (*d*_14_) of Samples Obtained under High 3D Printing Speed (100 mm/s) for Comparison^a^

	*X*_c_ (%)	*f*_c_	ε	*d*_14_ (pC/N)
sample 2	4.8	0.95	1.40	
sample 4	32.9	0.54	1.85	1.1
sample 6	73.4	0	2.45	0.1
optimized	57.1	0.91	3.00	8.5

a Sample 2 was 3D-printed on a platform at RT; sample 4 was 3D-printed on a platform at 60 °C and annealed at 100 °C for 10 min; sample 6 was 3D-printed on a platform at 90 °C and annealed at 120 °C for 30 min; and the optimized sample was 3D-printed on a platform at 60 °C and annealed at 100 °C for 10 min.

Overall, results clearly indicate that high printing speed induces higher polymer stretching and thus chain alignment along a specific direction during flow through the nozzle. Indeed, SEM images of the cross sections of deposited filaments for samples 3 and 4 reveal significant differences in shape and aspect ratio (see Supporting Figure S6). Sample 3, printed at low speed (5 mm/s), exhibits a more rounded cross section, whereas sample 4, printed at a higher speed (100 mm/s), displays an elongated morphology with a significantly higher aspect ratio, indicating that increased printing speed enhances polymer stretching during extrusion. Besides, they also indicate that the printing bed must be moderately heated to reduce the cooling rate of molten material and foster crystallization, though a trade-off must be reached between crystallization and relaxation of the flow-induced chain orientation at high temperature. Taking all of these aspects into account, the postprinting annealing was modified from the conditions used for sample 4 (10 min at 100 °C), so that the treatment was prolonged to 30 min, aiming to increase crystallinity while preventing chain relaxation to obtain an improved piezoelectric chain morphology.

Figure 5a shows the first heating DSC curves of this optimized 3D-printed PLLA sample, where the only observable events are the small endothermic step corresponding to *T*_g_ at 62 °C and the double melting behavior as in sample 4 (thermal properties are summarized in Table 2). As intended, the crystallinity was increased to 57%, which is almost twice the value of sample 4. Besides, the 1D-WAXS pattern shows main reflections of α-form, that is, reflections (200)/(110) and (203) at 16.7 and 19.1°, respectively. However, the peak 116 appears at 24.7° as well as for samples 4 and 5 (Figure 3d). This indicates that the prolonged annealing time allows the coexistence of α- and α′-phases in this case. In addition, the 1D-WAXS diffraction pattern is shown in Figure 5b and 2D-WAXS pattern (inset of Figure 5b) reveals a highly preferred orientation of the crystalline phase (*f*_c_ = 0.91). Figure S7 shows a closer inspection of 1D-WAXS diffraction patterns of samples 4–6 and optimized, which confirms the coexistence of α and α’ phases. Note that the azimuthal distribution of this sample shows a main narrow peak for (110)/(200) diffraction planes along with a small shoulder at a higher angle (Figure 5c). This seems to indicate that this sample contains regions with different orientation axes and likely also a distinct degree of orientation. The origin of these distinct regions is unknown but must be related to the processing conditions. For instance, when the hot nozzle passes over a previously deposited filament, it is plausible that localized reheating and partial polymer diffusion may induce a secondary orientation of the junction region with a slight misorientation, leading to the observed shoulder in the azimuthal profile. This has an effect on the piezoelectric properties of the material since it causes partial isotropization. The increase in both crystallinity and orientation is directly related to the improved resolution of the azimuthal distribution. Results clearly showed a significant increase not only of crystallinity (from 33 to 57%) as expected for the optimized sample but also of preferred orientation *f*_c_ (from 0.54 to 0.91). Under the chosen printing conditions, the molten material is subjected to high stretching that increases the molecular chain orientation within the deposited filament while favoring cold crystallization with the postprinting annealing. This seems to indicate that cold crystallization starts in less ordered regions, while prolonged annealing at 100 °C promotes crystallization of the higher oriented amorphous part, achieving a larger *f*_c_ value. A similar behavior was reported for postannealed PLLA fibers spun.^17^ Less ordered regions could be ascribed to the more defected and thinner interfaces between printed filaments, where it is known that the reheating of molten material deposition increases chain relaxation. Nevertheless, to further understand the role and degree of orientation of the amorphous material, small-angle X-ray scattering (SAXS) experiments are needed, which can even provide a deeper insight into how the amorphous phase also contributes to the overall piezoelectric response.

**Figure 5:**
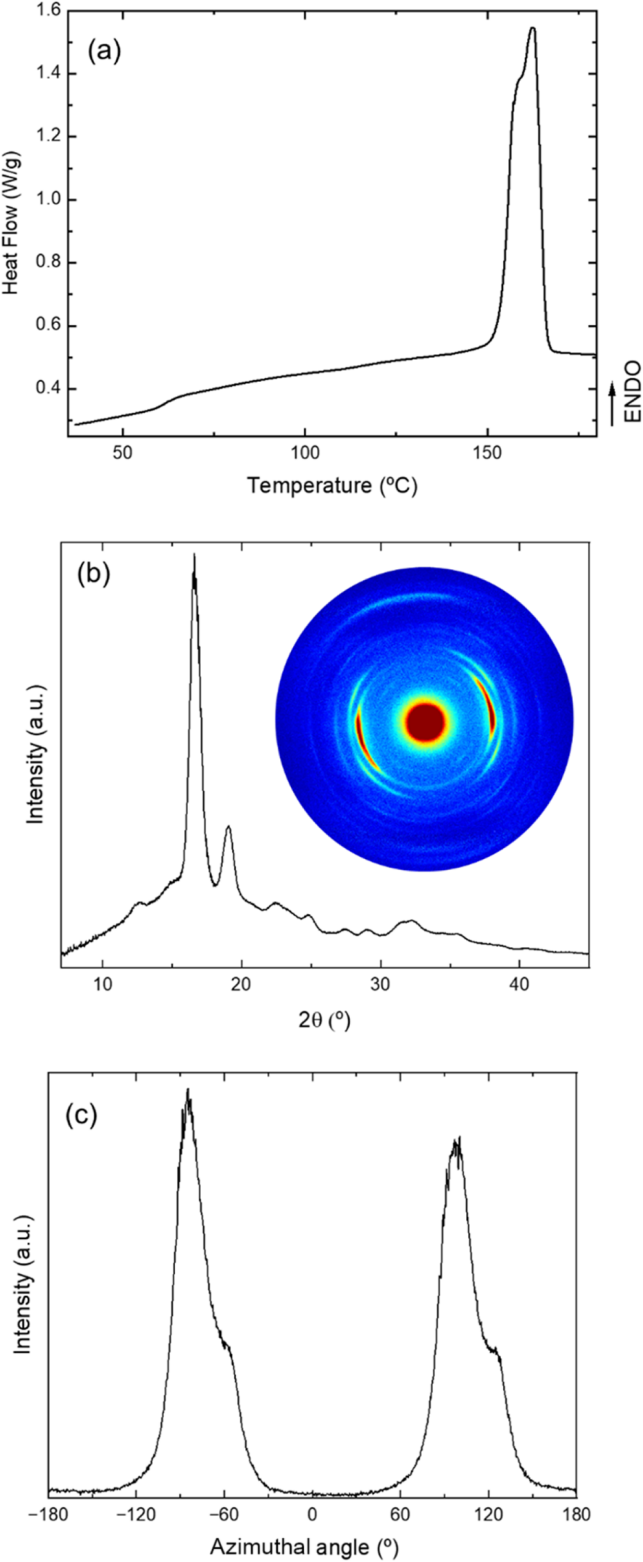
(a) Heat flow curve during 1st heating at 10 °C/min, along with (b) 1D-WAXS and (c) 2D-WAXS diffraction patterns corresponding to the optimized 3D-printed PLLA sample (printing speed of 100 mm/s on a platform at 60 °C and annealing at 90 °C for 30 min).

Dielectric properties of the 3D-printed PLLA samples are shown in Figure 6. Temperature dependences of the real (ε′) and imaginary (ε″) components of permittivity at several frequencies between 1 kHz and 1 MHz are displayed for the optimized 3D-printed PLLA laminate in Figure 6a,b, respectively. An initial RT permittivity of about 3.0 and dielectric losses below 0.005 were found, in agreement with the literature.^60,61^ During the first heating and up to *T*_g_, permittivity hardly changes and does not show any major frequency dependence, as expected from the reduced molecular mobility of both amorphous and crystalline phases. Once *T*_g_ is surpassed, permittivity increases with temperature, and large-frequency dispersion appears. This is accompanied by a peak in losses that shifts toward higher temperature with increasing frequency and indicates the presence of a dielectric relaxation, typically associated with the dynamic glass transition of the polymer chains,^62^ as the amorphous fraction evolves from a more rigid glass structure to one with enhanced molecular mobility.^63^

**Figure 6:**
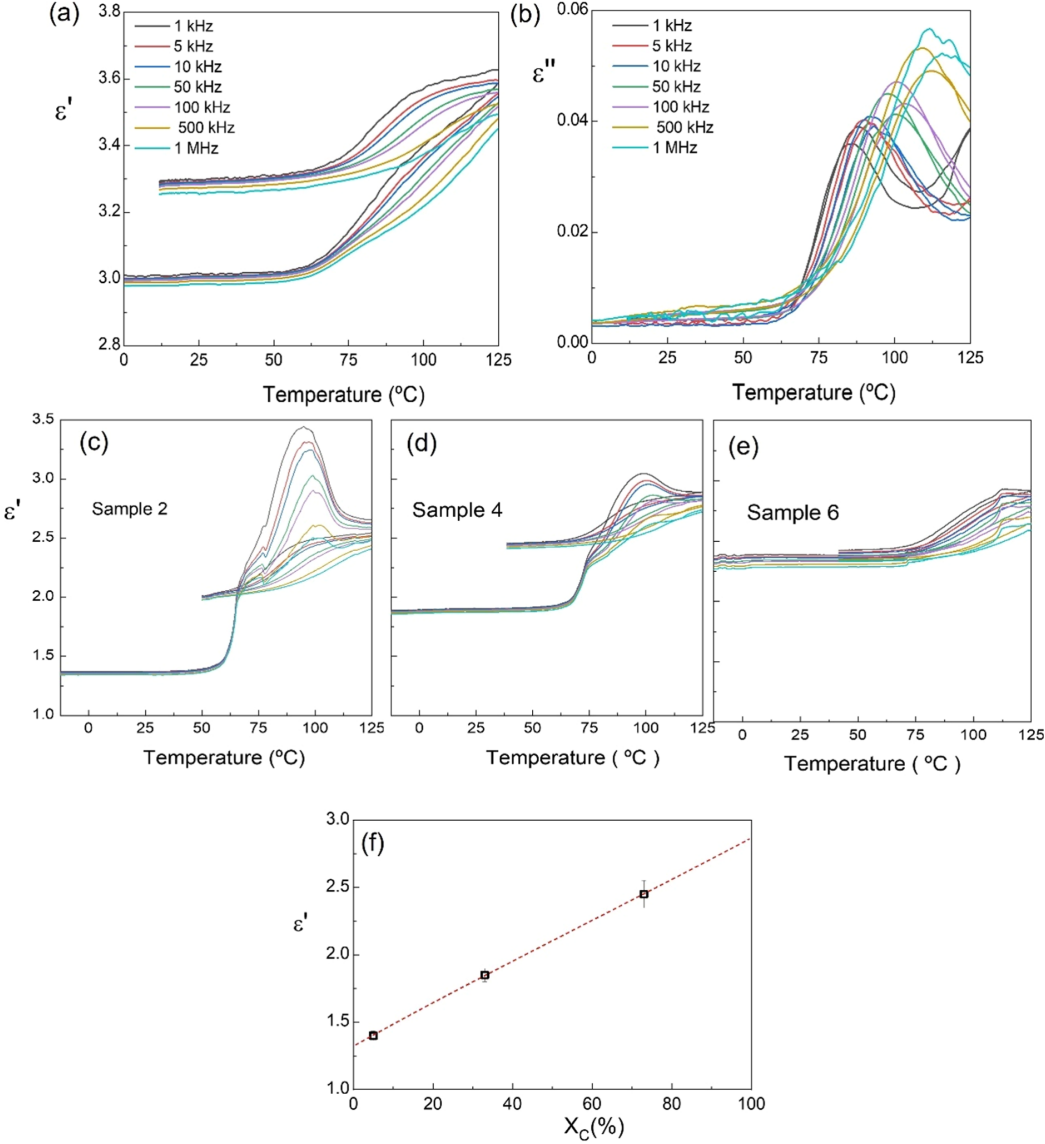
Temperature dependence of the (a) real (ε′) and (b) imaginary (ε″) components of permittivity during a heating/cooling cycle at different frequencies for optimized 3D-printed PLLA (printing speed of 100 mm/s on a platform at 60 °C and annealed at 90 °C for 30 min). Temperature dependence of the real (ε′) component of permittivity for (c) sample 2, 3D-printed on a platform at RT and 100 mm/s; (d) sample 4, 3D-printed on a platform at 60 °C,100 mm/s and annealed at 100 °C for 10 min; and (e) sample 6, 3D-printed on a platform at 90 °C, 100 mm/s and annealed at 120 °C for 30 min. (f) Linear relation between permittivity and the degree of crystallinity of these three samples.

Regarding the crystalline phase, it does not undergo a dielectric relaxation, yet its permittivity is higher than that of the amorphous one. This is clearly indicated by the temperature dependence of the real permittivity for samples 2, 4, and 6, which have different degrees of crystallinity, shown in Figure 6c–e. The same qualitative behavior is found, though permittivity values are different and closely correlated with the degree of crystallinity. In sample 2, for instance, which is nearly amorphous (*X*_c_ below 5%), an abrupt increase of permittivity is observed right above *T*_g_ during heating, followed by a broad dispersive maximum. This anomaly is certainly related to the cold crystallization process described in the DSC curves. Note that permittivity was measured at 2 °C/min instead of 10 °C/min used in DSC, so that a change in crystallization rate and a shift to a lower temperature can be expected. On cooling, however, curves showed the typical dielectric relaxation associated with the glass transition. Thus, as a result of the cold crystallization process taking place during the measurement, an increase in the RT permittivity from the initial value of about 1.4–2.0 was obtained, confirming the different permittivity values for the amorphous and crystalline states. A similar dielectric behavior was found in sample 4, in which the higher RT crystallinity (about 33%) yields a higher initial permittivity. The anomaly related to the cold crystallization is also observed in the heating curve, although less pronounced, in good agreement with DSC results, while the typical dielectric relaxation associated with glass transition is again observed on cooling. Finally, the dielectric curves of sample 6 are far more reversible, with little evidence of cold crystallization during measurement, as in the DSC curve, confirming the previous assertion. Considering the value of initial permittivity and the degree of crystallinity of these three samples, it is possible to deduce the values corresponding to the pure amorphous and crystalline states for this specific PLLA, as shown in Figure 6f. This information, rarely mentioned in the literature, could be of great importance to properly determine the piezocharge coefficient *d*_14_ when measuring open-circuit voltage response, from which piezovoltage coefficients *g*_13_ are obtained.^47^ Permittivity is often ignored in the piezoelectric characterization, and the assumption of literature data can lead to large errors. Besides, dielectric permittivity itself is a relevant parameter of piezoelectric materials and low values are advantageous for sensing and energy harvesting applications, which require high-voltage response.^27,64^

Figure 7a shows the open-circuit voltage generated under a cycling uniaxial force of 1 N at 1 Hz for three 3D-printed samples with different degrees of crystallinity and orientation factor but similar dimensions. Sample 6, which proved to be very crystalline (73%) but highly isotropic (*f*_c_ ∼ 0), exhibits the lowest piezovoltage signal (below 20 mV peak-to-peak), while sample 4, whose crystallinity is only 33% but shows certain molecular chain orientation (*f*_c_ = 0.54), exhibits an order of magnitude higher piezovoltage response (0.2 V peak-to-peak). This points to the key role of crystal orientation in the piezoelectric response of PLLA laminates, as anticipated. Consistently, the largest voltage was achieved in the optimized 3D-printed PLLA laminate with very high *f*_c_ = 0.91, near to 2 V peak-to-peak under 1 N and 1 Hz. The open-circuit voltage of this sample under increasing cycling uniaxial force is given in Figure 7b, from which a linear relationship can be established.

**Figure 7:**
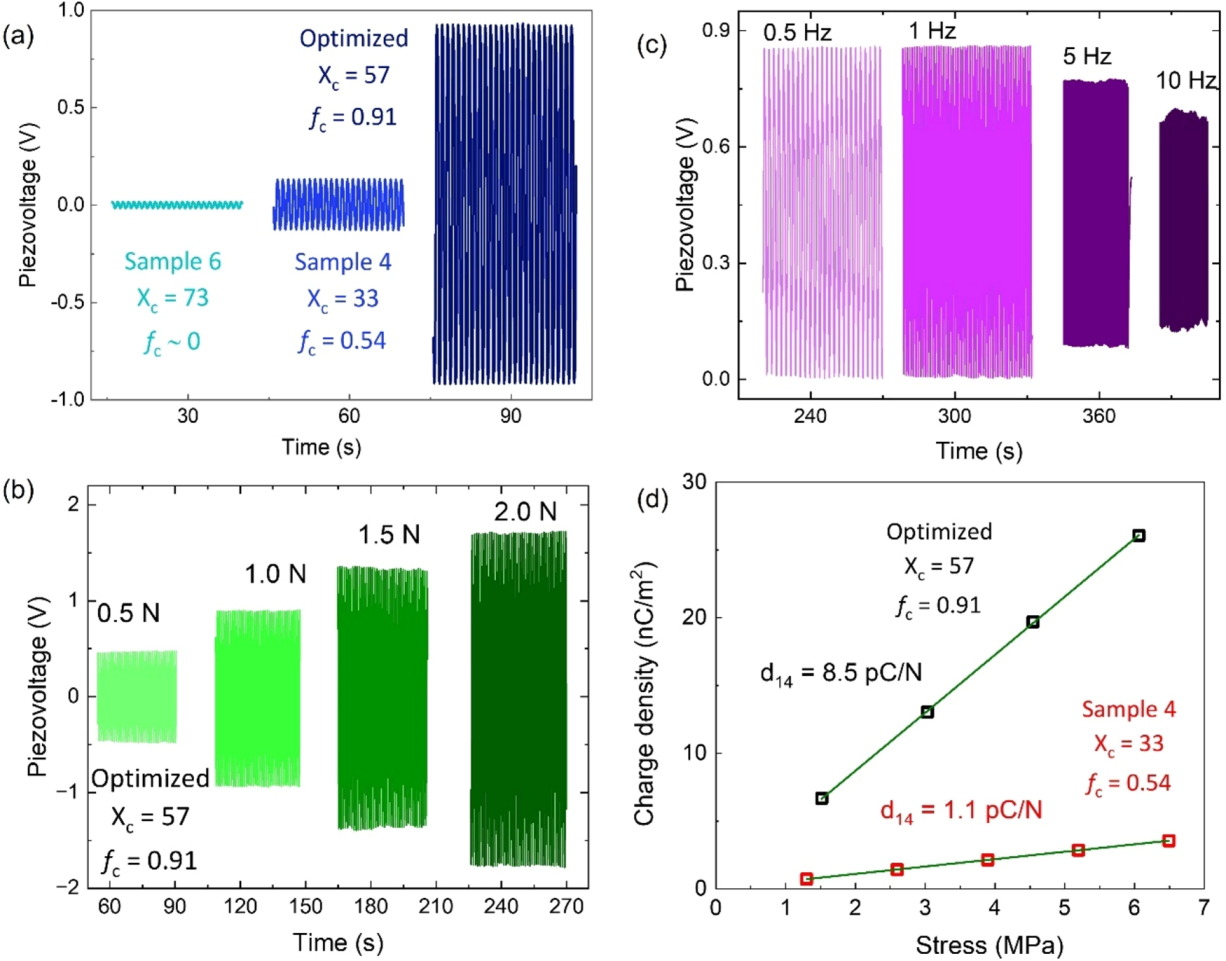
Open-circuit voltage under a cycling uniaxial force of 1 N (1 Hz) for three samples with different degrees of crystallinity and orientation factor. (b) Raw data of the generated piezovoltage at increasing cycling uniaxial force for optimized 3D-printed PLLA. (c) Frequency dependence in the range of 0.5–10 Hz of the open-circuit voltage response under a cycling uniaxial force of 0.5 N. (d) Linear dependence of the induced charge density *versus* applied stress to calculate the shear piezoelectric coefficient *d*_14_.

Figure 7c shows the frequency dependence in the range of 0.5–10 Hz of the open-circuit voltage response under a cycling uniaxial force of 0.5 N. No differences were found between 0.5 and 1 Hz, but above 1 Hz, the output voltage decreases, resulting in half the value at 10 Hz as compared to that at 0.5–1 Hz. Open-circuit voltage measurements are key to characterizing the performance of piezoelectric harvesters, where the device must be connected to a resistor load. Of course, the equivalent RC circuit of the polymer material must be considered in these measurements. In PLLA, permittivity is very low, and impedance is quite high, which significantly influences charge transport through the material during stretching cycles without reaching equilibrium. Besides, to characterize the piezoelectric coefficients, measurements should preferably be carried out in short-circuit.

Figure 7d shows this linear dependence of the short-circuit charge density generated against the applied stress (1 Hz), which is used to calculate the shear piezocoefficient *d*_14_. The optimized PLLA results in a very high *d*_14_ of 8.5 pC/N, which is among the best values reported for PLLA.^14,29,37–39^ It is known that the piezoelectricity of polylactides depends not only on the molecular chain orientation and crystallinity but also on the optical purity (amount of d-isomer) in the material. Shearing of aligned PLLA chains of the same chirality is impeded by the presence of a d-isomer, leading to the loss of piezoelectric response. So that larger piezoelectric performance would be expected by using PLLA of higher optical purity, although their use is quite challenging and much more expensive.^65^ Nevertheless, the results here clearly demonstrate that FDM is a viable technique to directly generate tailorable piezoelectric morphology in PLLA, opening up an interesting range of opportunities like the possibility of creating piezoelectric patterns within complex structures. Table 2 summarizes the values of crystallinity, Hermans orientation factor, dielectric permittivity, and piezoelectric coefficient *d*_14_ of samples 3D-printed at high speed (samples 2, 4, 6, and the optimized).

Despite the general acceptance of biocompatibility for PLLA, the specific chemical composition and purity of any commercial variant, including the one used in this study, must be rigorously assessed to ensure its biocompatibility, since it might contain toxic additives or contaminants incorporated during processing that could be harmful to users. For such a reason, we explored the biological responses of murine L929 fibroblasts, a conventional cell line for the biocompatibility assessment of biomaterials, of the optimized 3D-printed PLLA laminate. First, the morphological examination of the cells grown on the piezoelectric PLLA revealed a spindle-like morphology (size and shape) similar to that of cells cultured on glass substrates (control) (Figure 8a). However, cells on PLLA tended to be more frequently elongated and aligned on the 3D printing paths of deposited materials (marked with yellow arrows in Figure 8b). A closer inspection of the cells is shown in Figure 8c. This result is not surprising, as the promotion of cell alignment is already reported in 3D-printed tracks^66^ and has great potential to control metabolic and biological events within the body.^67,68^ It is unknown whether local piezoelectric responses of oriented polymer dipoles may also intervene in cell alignment, so further studies would be needed to clarify their contribution. The detection of filipodia and lamellipodia (indicated with red and blue arrows, respectively, in Figure 8d) confirms a good adhesion of this cell type on 3D-printed PLLA laminate. Regarding viability (Figure 8e–h), percentages of alive (measured in area) and dead cells (measured in number) demonstrated high viability in PLLA substrates, comparable to that on control substrates (glass).

**Figure 8:**
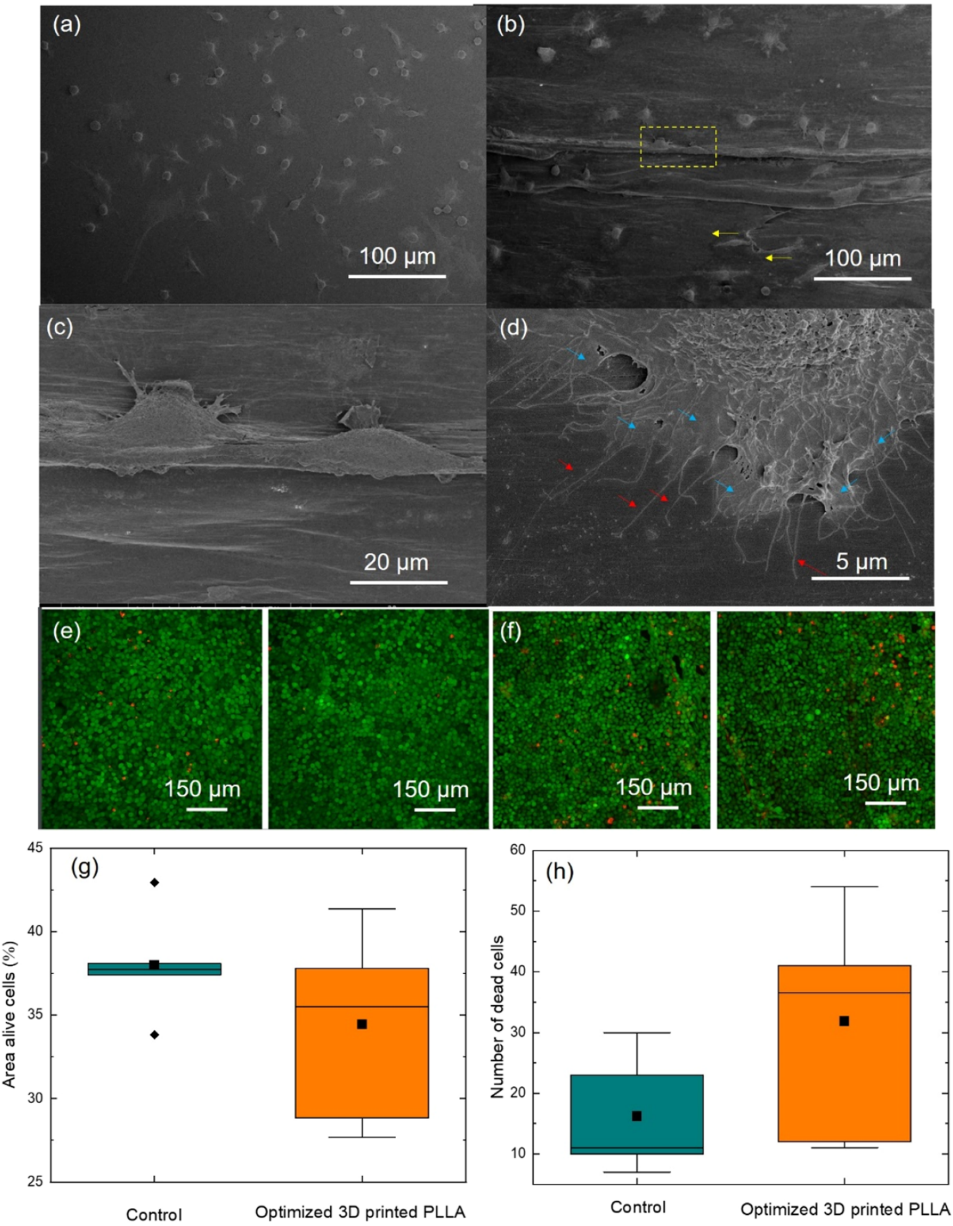
SEM images of the direct test showing cells on the surface of the samples. (a) Glass coverslip (control sample) after 3 days in culture; (b–d) optimized 3D-printed PLLA at different magnifications after 3 days in culture. Representative confocal images of control (e) and optimized 3D-printed PLLA (f) for viability screening after 7 days in culture. Viability quantification for the area of alive cells (g) and the number of dead cells (h) (*n* = 5 for control and *n* = 6 for PLLA). Cells that are alive are stained green (calcein), while dead ones are labeled red (ethidium homodimer-1).

Biomaterials that promote cell orientation offer significant advantages in regenerative medicine. Over the past decade, numerous studies have highlighted the beneficial role of cell alignment in tissue engineering. For instance, the oriented growth of myoblasts (muscle precursor cells) favored the formation of muscle fibers,^69^ while the alignment of fibroblasts is crucial for tendon and ligament development.^70^ Additionally, the aligned growth of neurons has been demonstrated to enhance nerve regeneration.^71^ Future work will focus on the exploration of electroactive cell types to deepen the applicability of these responsive materials in tissue engineering, the mechanisms underlying the cell responses found, and the evaluation of piezoelectric performance as part of complex 3D structures including *in vivo* degradation over time.

## 4 Conclusions

The crystalline fraction and phases of PLLA, as well as chain orientation, are tailorable through the 3D printing parameters in FDM, so that a range of piezoelectric chain morphologies can be obtained on demand. The technique is thus a powerful means of directly obtaining biocompatible piezoelectric platforms that are potentially capable of realizing piezoresponse complex patterns. One key parameter to foster piezoelectricity is fast printing speed, namely, up to 100 m/s that is significantly higher than those commonly used for PLA (40–60 mm/s). Polymer chains with a preferred orientation can be reproducibly obtained. A second key parameter is the bed temperature, which determines the cooling rate of the molten material when it is deposited. A trade-off must be reached in this case, so that chain mobility and, consequently, crystallization from melt are promoted without allowing significant relaxation. A value of 60 °C, which coincides with that normally recommended for PLA, was found adequate. The last relevant parameters are the temperature and time of an *in situ* postprinting annealing. This treatment aims at enhancing the cold crystallization of preoriented polymer chains. Optimized piezoelectric chain morphologies were obtained by carrying out this annealing at 90 °C for 30 min, while tailored piezoelectric coefficients can be easily obtained by the controlled variation of the printing parameters. Optimized 3D-printed PLLA here prepared from a commercial fiber-grade polymer presented distinctively oriented semicrystalline morphology with the coexistence of α and α’ polymorphs and exhibited a high shear piezocoefficient *d*_14_ of 8.5 pC/N. Although it is not feasible to assess the specific contribution of each polymorph, results here indicate that a high piezoelectric response of PLLA is related to α/α′ phases, so it is not exclusive of the frustrated β phase, as mentioned in some previous works.^20,47^ The biocompatibility of the material was assessed with L929 fibroblasts, which proved to have good cell adhesion and high viability on these substrates after a 1 week culture. The obtained *in vitro* biological results illustrated the promotion of cell alignment along the 3D printing direction with cells that tended to present elongated morphologies more frequently than on control samples. These results highlight the potential of 3D-printed piezoelectric PLLA for biomedical applications (*e.g.*, tissue engineering or biomedical implants, among others). For example, bone piezoelectric coefficients under shear loads range *d*_14_ ∼ 0.7–2.9 pC/N,^72^ while that of wet bone reaches 8 pC/N.^73^ This means that the 3D-printed PLLA already meets the requirements for mimicking the piezoelectric behavior of bone.

## Supplementary Material



## Notes

Experimental setup; peak analysis of azimuthal profiles for Hermans orientation factor; closer inspection and comparison of 2D and 1D-WAXS diffraction patterns; and additional SEM images (PDF)
