# Supplementary material for: Tailorable Piezoelectric Chain Morphology in Biocompatible Poly‑l‑lactide Induced by Melt-Based 3D Printing
Source: ACS Appl Polym Mater. 2025 May 6;7(10):6067–81. doi: 10.1021/acsapm.5c00450 (PMC12107498; doi:10.1021/acsapm.5c00450)
Supplement: Supplementary file 1 [file ap5c00450_si_001.pdf]

Supporting Information

Tailorable piezoelectric chain morphology in biocompatible poly-L-lactide induced by melt-based 3D printing

Cristina Pascual-González[1*], Gustavo Pacheco-Carpio[1], Juan P. Fernández-Blázquez[3], María Concepción Serrano[1], Bernd Wicklein[1], Miguel Algueró[1] and Harvey Amorín[1]

[1]Instituto de Ciencia de Materiales de Madrid (ICMM), CSIC. C/ Sor Juana Inés de la Cruz 3, Cantoblanco, 28049 Madrid, Spain

[2] IMDEA Materials Institute, C/ Eric Kandel 2, Getafe, 28906, Madrid, Spain

Correspondence to: cristina.pascual@csic.es

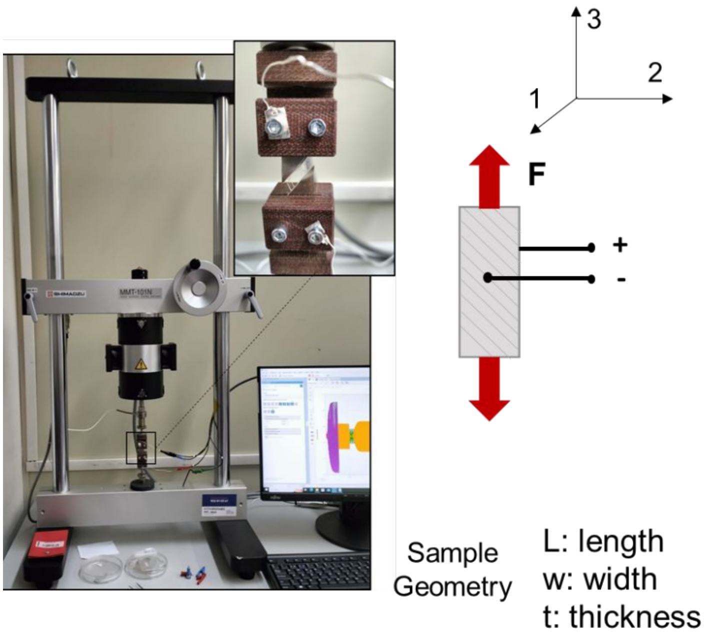

***Figure S1**: Schematic diagram of the sample and experimental setup for piezoelectric characterization.*

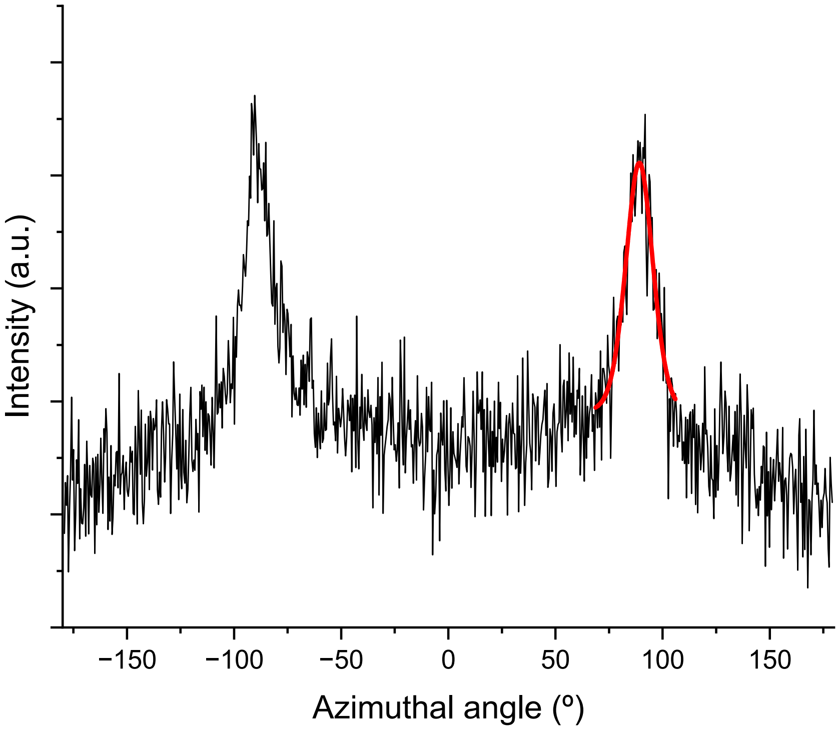

Model	PsdVoigt1
Equation	y = y0 + A * (mu * (2/pi) * (w / (4*(x-xc)^2 + w^2)) + (1 - mu) * (sqrt(4*ln(2)) / (sqrt(pi) * w)) * exp(-(4*ln(2)/w^2)*(x-xc)^2))
Plot	B
y0	39.85338 ± 0.33823
xc	89.15029 ± 0.28286
A	41.58517 ± 23.9789
w	15.12165 ± 1.69379
mu	0.39144 ± 0.87934
Reduced Chi-Sqr	0.10767
R-Square (COD)	0.84512
Adj. R-Square	0.83686

Figure S2: *Peak analysis of azimuthal profiles for Hermans orientation factor calculation of sample 2.*

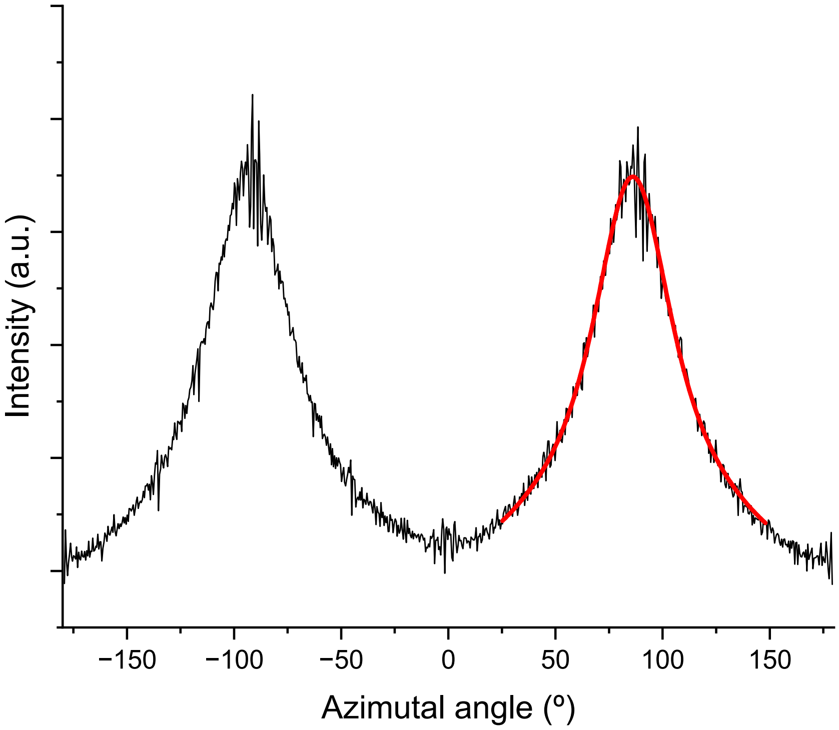

Model	PsdVoigt1
Equation	y = y0 + A * (mu * (2/pi) * (w / (4*(x-xc)^2 + w^2)) + (1 - mu) * (sqrt(4*ln(2)) / (sqrt(pi) * w)) * exp(-(4*ln(2)/w^2)*(x-xc)^2))
Plot	B
y0	47.06555 ± 2.9594
xc	86.08204 ± 0.12872
A	3415.76059 ± 801.36945
w	52.92734 ± 4.59778
mu	1.16734 ± 0.13938
Reduced Chi-Sqr	1.36705
R-Square (COD)	0.98656
Adj. R-Square	0.98635

Figure S3: *Peak analysis of azimuthal profiles for Hermans orientation factor calculation of sample 4.*

Peak Analysis

Data Set:[WAXSBuenaazi1]WAXS_Buena_azi_1D!B		Date:14/03/2025
BaseLine:Line		
Chi^2=4.71251E-01	Adj. R-Square=9.97307E-01	# of Data Points=224
SS=1.01319E+02	Degrees of Freedom=215	

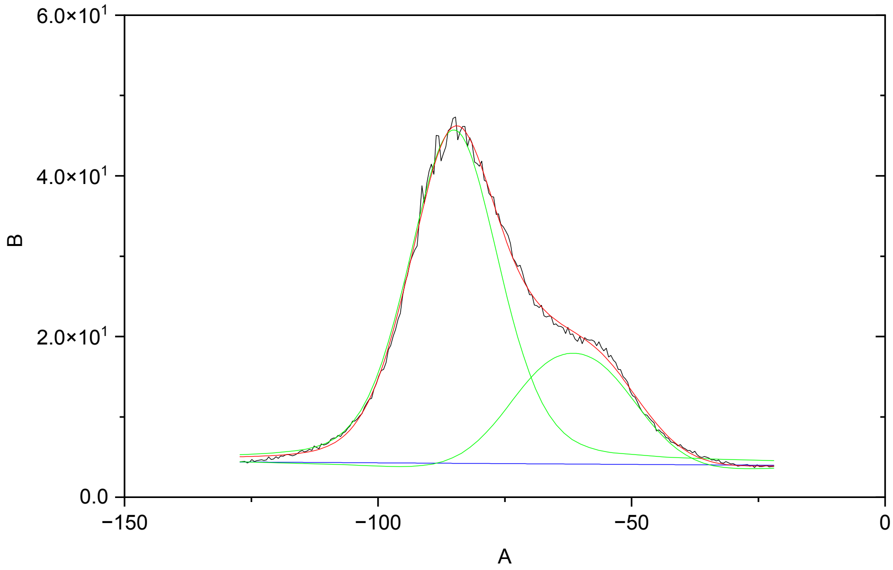

Fitting Results

Peak Index	Peak Type	Area Intg	FWHM	Max Height	Center Grvty	Area IntgP
1	PsdVoigt1	984.77705	20.8732	41.20404	−85.04334	74.60615
2	PsdVoigt1	328.05838	26.02123	13.50768	−61.49577	24.85352

Figure S4: *Peak analysis of azimuthal profiles for Hermans orientation factor calculation of an optimized sample.*

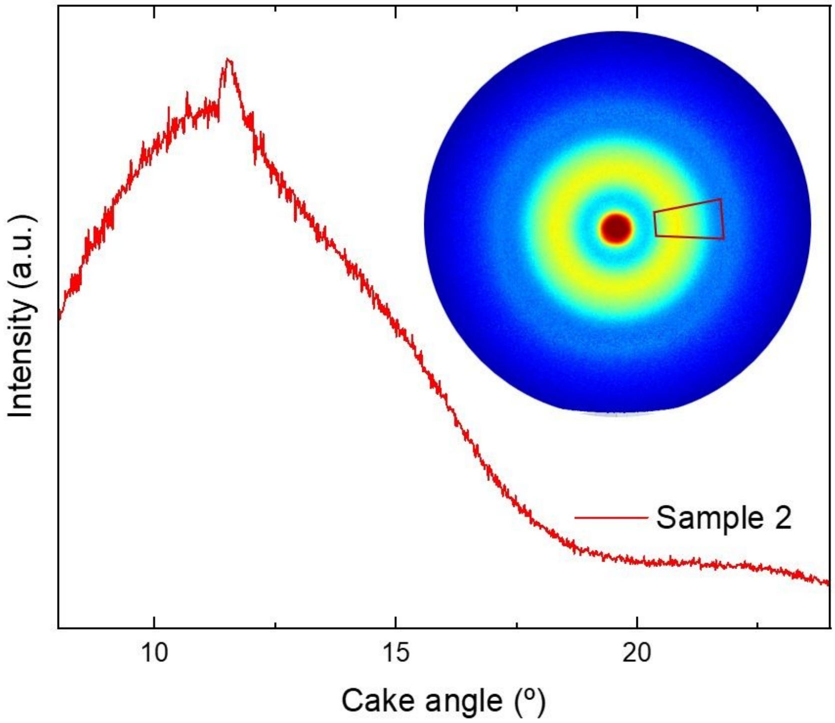

Figure S5: *Incipient crystallisation is detected in 1D-WAXS diffraction pattern of sample 2 corresponding to the area indicated in inset 2D-WAXS pattern.*

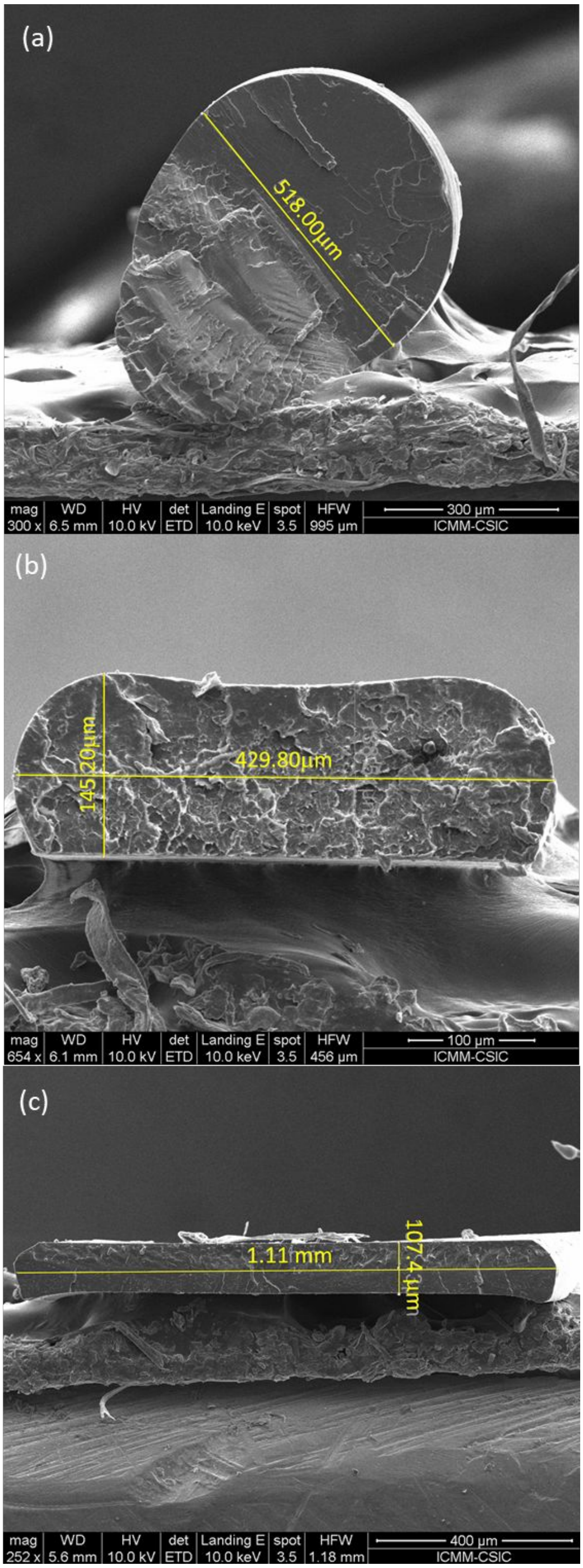

Figure S6: *Representative SEM images of cross sections of (a) the filament that goes through the nozzle, (b) deposited filament of sample 3 at slow 3D printing speed (5 mm/s) and (c) deposited filament of sample 4 at rapid 3D printing speed (100 mm/s).*

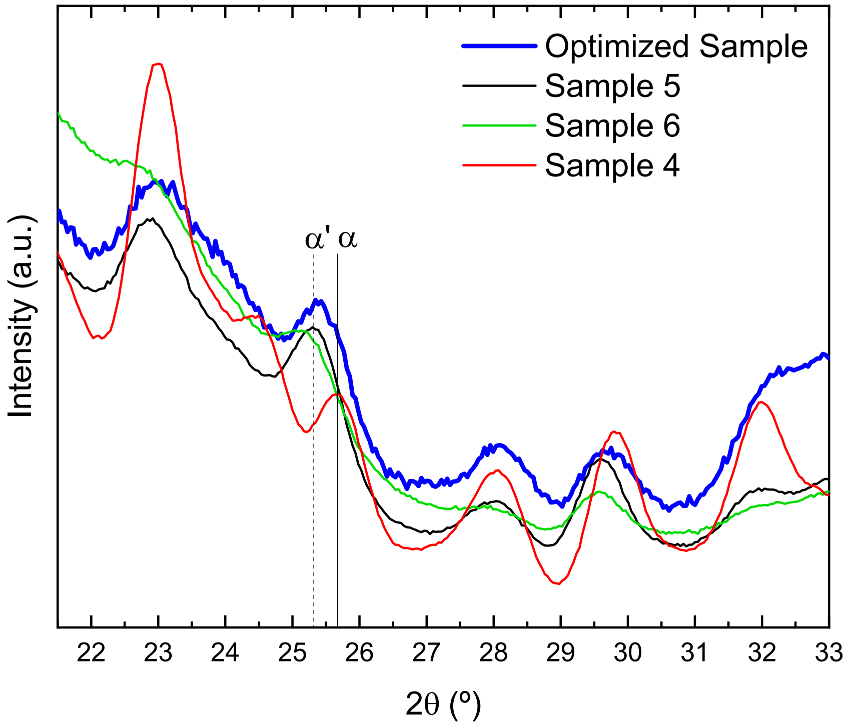

Figure S7: *Closer inspection of 1D-WAXS diffraction patterns of sample 4, 5, 6 and optimized. The area under the pattern is normalized. 1D-WAXS diffraction pattern of optimized sample is consistent with the coexistence of α and α' phases.*
